# ﻿*Oviculabiradiata*, a new genus of Compositae from Big Bend National Park in Trans-Pecos Texas

**DOI:** 10.3897/phytokeys.252.137624

**Published:** 2025-02-18

**Authors:** Debra L. Manley, Isaac H. Lichter Marck, Keily Peralta, Arturo Castro Castro, Kelsey A. Wogan, Carolyn V. Whiting, A. Michael Powell

**Affiliations:** 1 Science and Resource Management, Big Bend National Park, PO Box 129, Big Bend National Park, TX 79834-0129, USA Science and Resource Management, Big Bend National Park Big Bend National Park United States of America; 2 Department of Botany, Institute for Biodiversity Science and Sustainability, California Academy of Sciences, 55 Music Concourse Dr., San Francisco, CA 94118, USA Institute for Biodiversity Science and Sustainability, California Academy of Sciences San Francisco United States of America; 3 Centro Interdisciplinario de Investigación para el Desarrollo Integral Regional, Sigma 119, Fracc. 20 de Noviembre II, Durango, Mexico Centro Interdisciplinario de Investigación para el Desarrollo Integral Regional Durango Mexico; 4 Department of Natural Sciences, Sul Ross State University, East Highway 90, Alpine, TX 79832, USA Sul Ross State University Alpine United States of America

**Keywords:** Asteraceae, biodiversity, calciphile, Chihuahuan Desert, Helenieae, taxonomy, Tetraneurinae, Asteraceae, biodiversidad, calcifilo, Desierto Chihuahuense, Helenieae, taxonomía, Tetraneurinae

## Abstract

Here, we describe and illustrate a new monospecific genus of Compositae, *Oviculabiradiata***gen. et sp. nov.**, from the Chihuahuan Desert in Big Bend National Park, Texas. *Oviculabiradiata* is a very locally abundant, yet range-limited, spring annual herb found in coarse calcareous alluvium. Based on its pistillate ray florets, pappus of hyaline, aristate scales, tomentose foliage and slightly saucer-shaped to flat, epaleate receptacle, we determine that the new species has affinities with the Helenioid subtribe Tetraneurinae in the Heliantheae alliance. Molecular phylogenetic analysis of nrDNA (ITS) sequence data supports the phylogenetic position of *Oviculabiradiata* within the subtribe Tetranuerinae, where it is resolved as the sister lineage to the genus *Psilostrophe*. We also present detailed habitat information, high-resolution images captured using a dissecting microscope and scanning electron micrographs of vegetative and reproductive characters of *Oviculabiradiata* and related taxa, as well as provide an updated key to the genera of Tetraneurinae. Finally, we discuss the significance of this remarkable discovery for community science, biodiversity exploration and plant conservation in the Chihuahuan Desert.

## Abstract (es)

Se ilustra y describe un nuevo género monoespecífico de Compositae, *Oviculabiradiata***gen. et sp. nov.**, del Desierto Chihuahuense en el Parque Nacional Big Bend, Texas. *Oviculabiradiata* es una hierba annual muy localmente abundante, pero con un rango limitado, florece en la primavera y se encuentra en aluviones calcáreos gruesos. Con base en sus flores pistiladas con corola radiada, vilano de escamas aristadas y hialinas, follaje tomentoso y receptáculo ligeramente en forma de platillo a plano y epaleado, determinamos que la nueva especie tiene afinidades con Helenieae subtribu Tetraneurinae, en la alianza Heliantheae. Análisis cladísticos moleculares de secuencias del ADN ribosomal nuclear (ITS) apoyan la posición filogenética de *O.biradiata* dentro de la subtribu Tetraneurinae, donde resulta el lineage hermano del género *Psilostrophe*. También presentamos información detallada sobre el hábitat, imágenes de alta resolución usando un microscopio de disección y micrografías electrónicas de barrido de caracteres vegetativos y reproductivos de *O.biradiata* y taxa relacionados, así como una clave actualizada para los géneros de Tetranuerinae. Finalmente, discutimos la significancia del descubrimiento en relación a la ciencia ciudadana, exploración de biodiversidad, y la conservación de plantas en el Desierto Chihuahuense.

## ﻿Introduction

The Chihuahuan Desert is the largest and most biologically diverse warm desert in North America (Bell et al. 2014). Big Bend National Park is in southern Brewster County, Texas, bordered to the south by the Rio Grande. Its 801,165 acres (324,220 hectares) include some of the best representation of Chihuahuan Desert microhabitat diversity in the United States, including within the Chisos Mountain range (highest elevation ca. 7825 ft. (2385 m)) and numerous smaller peaks separated by low desert bajadas. Eighty-nine plant species of conservation concern are found in the park (Louie 1996; Poole et al. 2007, Texas Parks and Wildlife Department 2024). Many of these species of concern have limited distributions that extend into adjacent areas in Mexico or into Chihuahuan Desert habitats to the immediate north and east of the park boundary (Powell and Worthington 2018).

Previous floristic and wildflower studies centred in and near Big Bend National Park, include McDougall and Sperry (1951), Warnock (1970, 1974, 1977), Fenstermacher et al. (2008), Morey (2008, 2024), Hardy (2009), Weckesser and Terry (2014) and Powell and Worthington (2018). Wauer (1973, 1997) explored many off-the-trail areas searching for plants and animals of natural history interest. Potentially relevant floristic treatises of wider range include Correll and Johnston (1970), Henrickson et al. (1997), Turner et al. (2003), Turner (2013), Eason (2018) and Allred et al. (2020). Even though the park has been rather thoroughly botanised in the vicinity of most accessible areas, additional new plant discoveries are possible because of its extensive habitat diversity in stretches of remote terrain.

**Figure 1.**
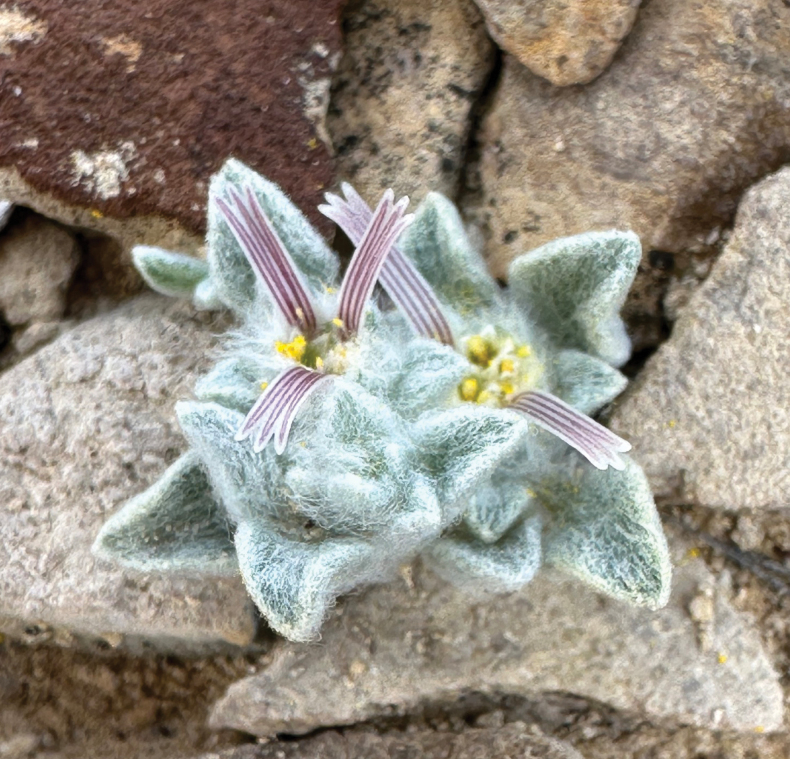
First photograph of *Oviculabiradiata* taken by Deb Manley on 2 March 2024.

**Figure 2.**
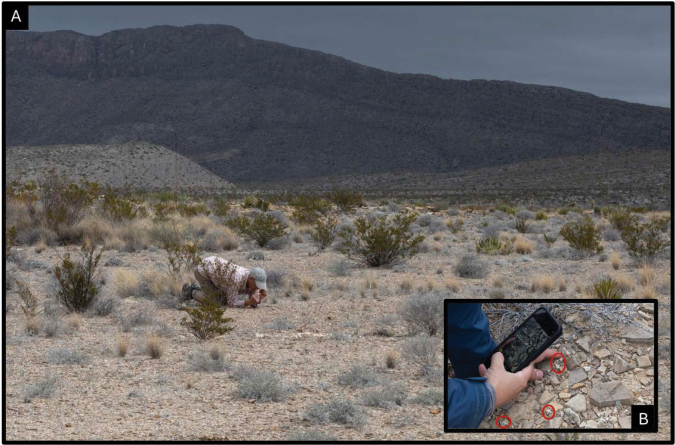
Researchers examining individuals in habitat. **A** Patty Manning scanning the ground in appropriate habitat for individuals of *Oviculabiradiata***B** NPS botanist Carolyn Whiting photographing *O.biradiata* (circled in red). Photos by Cathy Hoyt on 20 April 2024.

**Figure 3.**
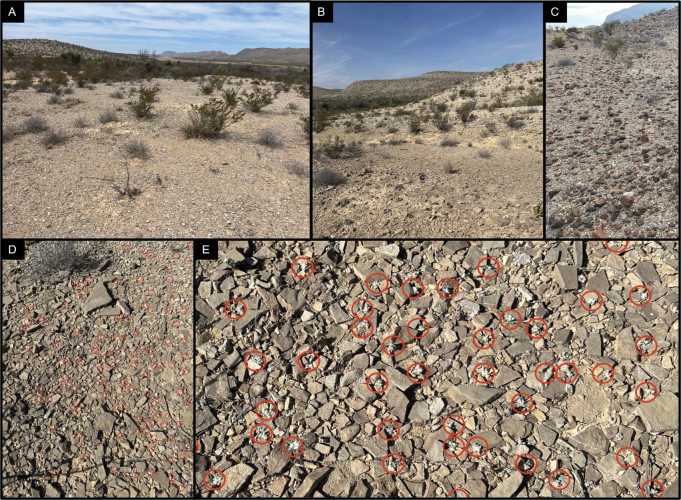
Known habitat of *Oviculabiradiata*. **A, B** Habitat with evident pediment slopes **C** slight habitat variation with iron-bearing rocks present in calcareous cobbles **D** overview of population locality with individual plants circled in red **E** close-up of individual plants in habitat illustrating cryptic appearance amongst calcareous surficial deposits. Photos by James Bailey (**A**) in April 2024 and Deb Manley on 20 April 2024 (**B–E**).

**Figure 4.**
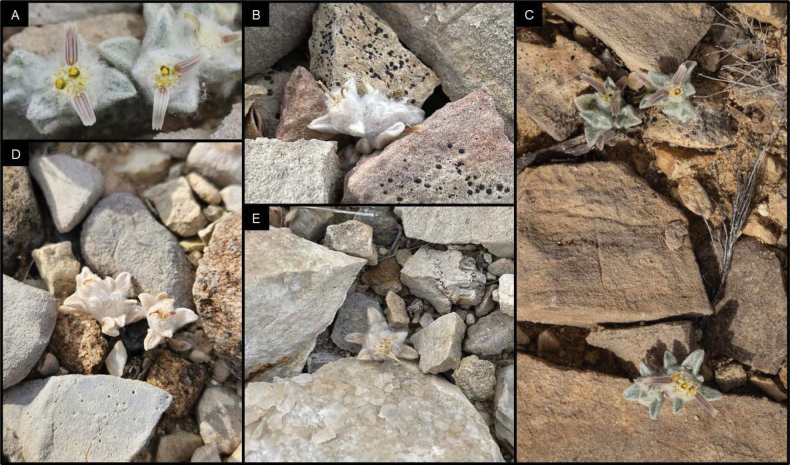
Images of *Oviculabiradiata* individuals representing the “small” growth habit that occurs most frequently in all three known locations. Photographs by James Bailey in April 2024 (**A**), Kelsey Wogan on 27 April 2024 (**B, E**), Cathy Hoyt on 2 March 2024 (**C**), Dana Sloan on 27 April 2024 (**D**).

**Figure 5.**
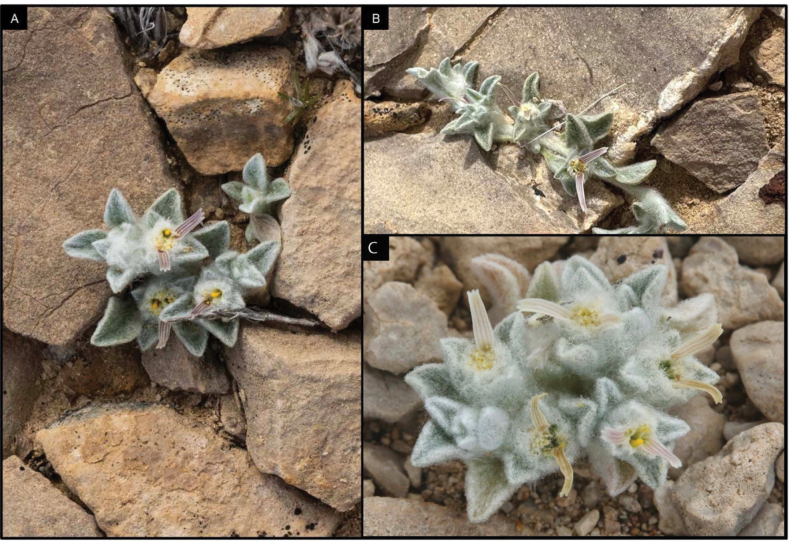
Examples of moderately sized individuals found occasionally throughout the known locations. Photographs by Cathy Hoyt (**A)** & Deb Manley (**B**) on 2 March 2024 and James Bailey in April 2024 (**C**).

**Figure 6.**
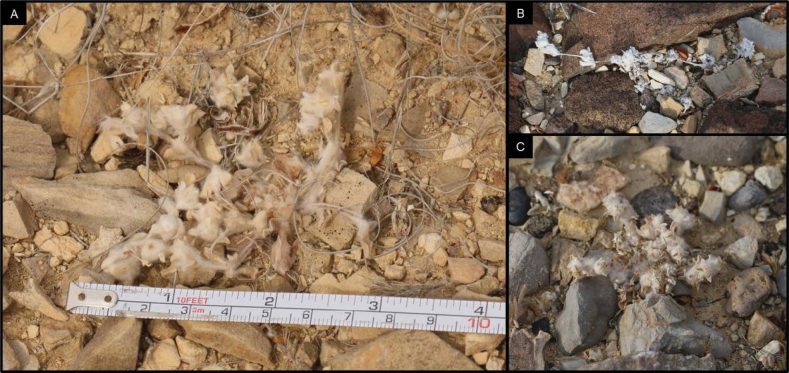
Largest individuals of *Oviculabiradiata* encountered by researchers in known localities thus far. Photographs by Deb Manley (**A, B**) on 20 April 2024 & A. Michael Powell (**C**) on 27 April 2024.

**Figure 7.**
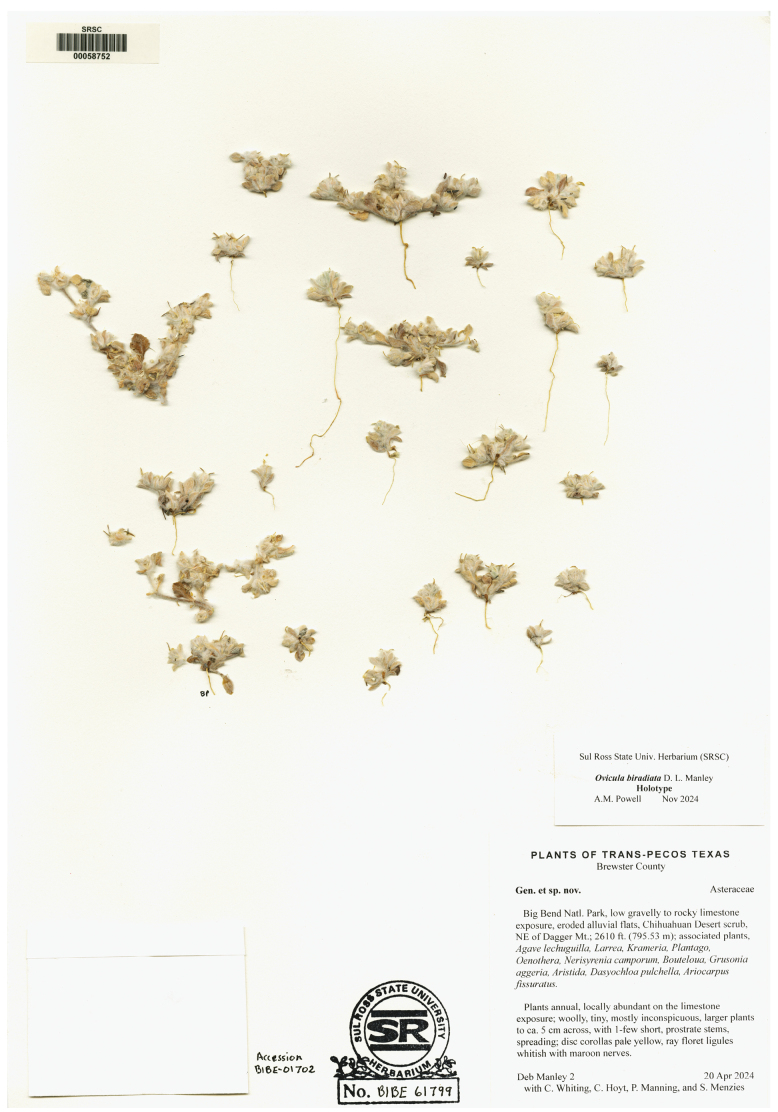
Scan of the Holotype of *Oviculabiradiata*.

**Figure 8.**
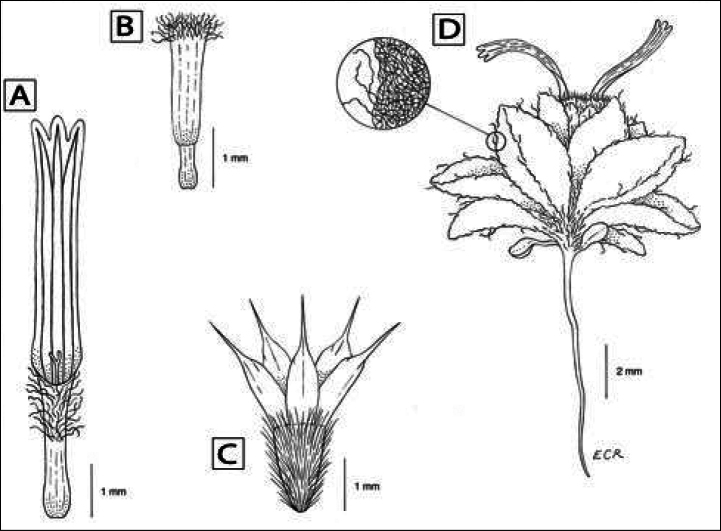
Line drawing of *Oviculabiradiata* gen. et. sp. nov. **A** Ray floret without cypsela **B** disc floret without cypsela **C** cypsela **D** habit with close up of leaf surface illustrating nature of indumentum. Illustration by Ellen Ruggia, based on material from the paratype (Manley 3).

**Figure 9.**
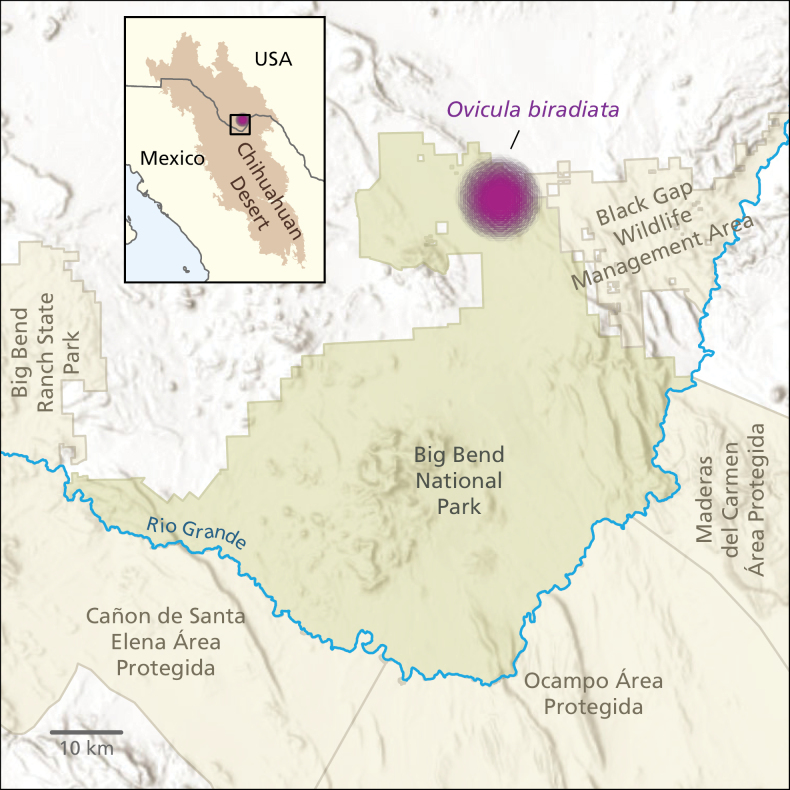
Approximate range map of *Oviculabiradiata*. Geographical location of the known range of *O.biradiata* in Big Bend National Park in Brewster County, Texas. The boundaries of the park are illustrated in dark green. The purple dot marks the approximate area where three sub populations, each with abundant individuals of *O.biradiata*, were located. An inset illustrates the location of the study site within the Chihuahuan Desert.

On 2 March 2024, while traversing cross country in search of rare plant populations, the first author photographed an anomalous composite and posted images on iNaturalist. These diminutive plants, observed during the peak of their growing season, were inconspicuous annuals, from less than one centimetre to 3–7 centimetres across, prostrate and densely white-woolly, matching the whitish colour of their calcareous gravel substrate (Figs 1–9). Following a review, Park authorities granted us permission to collect a few individuals for further study. From the limited material on hand and the photos we had taken, we were able to discern characters suggestive of relationships to the tribe Helenieae Lindl. (sensu Baldwin et al. (2002)), especially *Tetraneuris* Greene. These characters included obconic fruits with five paleaceous, aristate scales and pistillate ray florets with maroon linear markings (Bierner and Turner 2006; Funk et al. 2009; Spellenberg and Zucker 2019). To test the hypothesised relationship to Helenieae, we carried out a more detailed study of inflorescence and fruit characters using scanning electron microscopy and sequenced nrDNA sequence data for one DNA gene region, the Internal Transcribed Spacer (ITS). Here, we present morphological, micro-anatomical and molecular phylogenetic evidence that supports description of this plant as a new genus and species of subtribe Tetraneurinae Rydb.

## ﻿Methods

### ﻿Field and herbarium collections

After the National Park Service granted a research permit for this study (BIBE-2024-SCI-0015), plants were collected from two field locations, briefly dried and deposited in the A. Michael Powell (SRSC) and California Academy of Sciences (CAS) Herbaria for mounting and further study. To our knowledge, besides these collections, this previously unknown species has not been deposited in herbaria before. Dried vegetative and reproductive material of putative close relatives was obtained for detailed morphological study and DNA sequencing from herbarium specimens at SRSU and CAS. Sampling included representatives of genera in subtribe Tetraneurinae, i.e. *Amblyolepis* DC., *Baileya* Harv. & A. Gray ex Torr., *Hymenoxys* Cass., *Psilostrophe* DC. and *Tetraneuris* Greene. A complete list of specimens sampled and GenBank accession numbers is presented in Table 1.

**Table 1.** Specimen voucher data and GenBank accession numbers for herbarium material used in molecular phylogenetic analyses and scanning electron microscopy.

Taxon	Purpose	Accession number	Collector	Collector number	Date	GenBank accession number
*Psilostrophebakeri* Greene	DNA/SEM	CAS 818281	A. Cronsquist	11645	13 June 1980	PQ144335
*Baileyapauciradiata* Harv. & A. Gray	SEM	CAS 288097	H.S. Gentry		25 Feb 1933	
Tetraneurisacaulis(Greene)K.F. Parkervar.arizonica	SEM	CAS 731062	J. Henrickson	10576	4 June 1973	
*Psilostrophesparsiflora* (A.Gray) Nelson	DNA	CAS 5935742	M. Butterwick	7526	19 May 1981	PQ144336
*Tetraneurisscaposa* (DC.) Greene	DNA/SEM	CAS 700109	B. Turner	15128	5 June 1983	PQ144339
*Baileyamultiradiata* harv. & A. Gray	SEM	CAS 713832	P. Munz	13688	3 May 1935	
*Amblyolepissetigera* DC.	SEM	CAS 765096	B. Ertter	5598	13 March 1985	
*Psilostrophevillosa* Rydb. ex Britton	DNA	CAS 507562	P. Raven	19297	7 June 1964	PQ144334
HymenoxyscooperiCockerellvar.cooperi	SEM	CAS 1005608	J. Henrickson	10521	4 June 1973	
*Psilostrophemexicana* R.C. Br.	DNA	CAS 720959	J.L. Villaseñor	1591	23 September 1982	PQ144337
*Psilostrophegnaphalodes* DC.	DNA	CAS 701425	S. Sunderberg	1214	15 August 1981	PQ144338
*Oviculabiradiata* Manley	DNA/SEM	SRSC 00058752	D. Manley	2	17 April 2024	PQ144333

### ﻿Morphological study

We examined morphological characters from field collections of the new species and exsiccate of putative close relatives using dissecting microscopy. Images of microscopic features were captured using a Leica M60 stereomicroscope (Leica Camera, Wetztlar, Germany) outfitted with a digital camera. Morphology was compared with representatives of all recognised genera of tribe Helenieae (Table 2). In addition, surface morphology of floral and vegetative structures was analysed and imaged using a Hitachi SU3500 Scanning Electron Microscope (SEM; Hitachi, Tokyo, Japan) at the California Academy of Sciences. Initially, inflorescence and fruit structures were disassembled under a dissecting microscope and loaded on to an 18 mm pin-mounted SEM stub using double sticky tape. To enhance the electron conductivity of samples, we then used a Cressington Sputter Coater 108 (Cressington, Watford, UK) at a vacuum pressure of 0.8 Pa to apply a 5 nm layer of gold-palladium to the sample for 50 seconds. We observed traits of potential phylogenetic informativeness following Robinson (1981) and King and Robinson (1970) at 15 kV and a working distance of 7 mm, under automated controls for focus, contrast and stigmation.

### ﻿DNA extraction, amplification and sequencing

Following removal of the woolly indumentum under a dissecting microscope, fresh, field-collected leaves were dried for one week using silica gel and pulverised in a Qiagen tissue lyser (Qiagen, inc., Valencia, California) with a mixture of zircon beads and autoclaved sand. Genomic DNA was extracted using the DNEasy plant mini-kit (Qiagen, inc., Valencia, California) in the Center for Comparative Genomics at the California Academy of Sciences. We followed the provided protocol with a modified incubation in a cell-lysis buffer extended to 16 hours. A Polymerase Chain Reaction (PCR) master mix containing 9.1 ul H_2_0, 0.3 ul DnTPs, 0.15 ul Taq polymerase, 0.75 ul MgCl_2_, 1.5 ul 10× PCR buffer and 0.6 ul Bovine serum Albumin (BVA) was combined with two primers for amplifying the Internal Transcribed Spacer region (ITS), ITS4 and LEU (White et al. 1990). Two ul of undiluted genomic DNA was combined with the PCR master mix and transferred to a thermal cycler programmed to the following conditions: 97 degrees for 1 min; 40 cycles of 97 degrees for 10 sec, 48 degrees for 30 sec, 72 degrees C for 20 seconds; and 72 degrees C for 7 minutes. Post-PCR products were checked for successful amplification using gel electrophoresis and unpurified PCR-product was forward and reverse Sanger sequenced by Genewiz (Azenta US Inc., Burlington, MA).

Following an initial search of the NCBI BLAST database to confirm a close match between our ITS sequence and putative closely-related taxa, we visually aligned the ITS sequence for the new species with the Baldwin et al. (2002) published data matrix for epaleate tribes of the Heliantheae alliance. Once we recovered strong evidence for the sister relationship of the new species with *Psilostrophe* in tribe Helenieae, we generated additional sequence data for all recognised minimum-rank taxa of *Psilostrophe* and *Tetraneurisscaposa* (DC.) Greene using leaf tissues sampled from herbarium specimens. For these additional samples, we followed an extraction and amplification procedure identical to that described above. Selection of a model of molecular substitution and Maximum Likelihood (ML) inference of a phylogenetic tree, based on aligned data matrices of ITS, was inferred using IQTREE2 (Minh et al. 2020) and bootstrap support for nodes was calculated, based on 1000 iterations using fast-bootstrapping.

## ﻿Results

### ﻿Phylogenetic relationships

**Figure 10.**
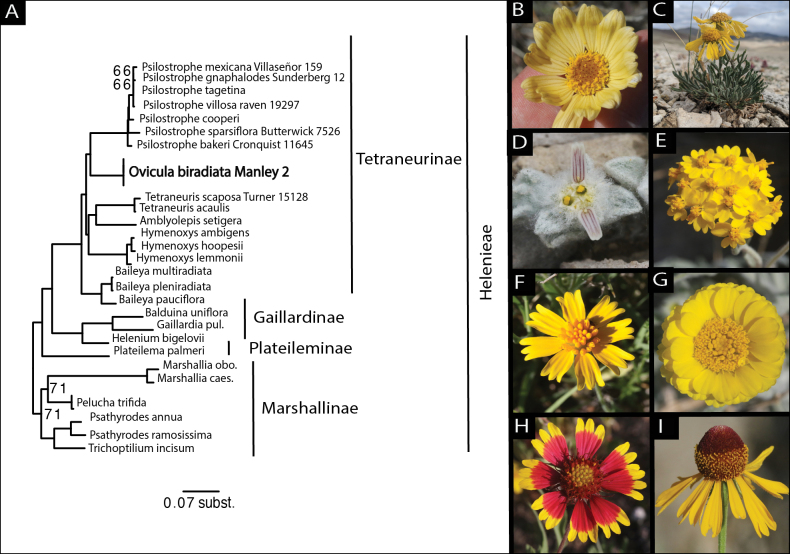
Phylogenetic relationships of *Oviculabiradiata* and representative photographs of genera of Helenieae. **A** Maximum Likelihood (ML) phylogenetic tree of Helenieae, based on an aligned matrix of nrDNA sequence data from the Internal Transcribed Spacer region. ITS sequences generated as part of this study have collector numbers indicated to the right. Subtribes are indicated with text. *Oviculabiradiata* is in bold **B***Tetraneurisscaposa***C***Hymenoxyscooperi***D***Oviculabiradiata***E***Psilostrophetagetina***F***Amblyolepissetigera***G***Baileyapleniradiata***H***Gaillardiapulchella***I***Heleniumamphibolum*. Photographs by Peri Lee Pipkin (**C**) and James Bailey (**B, D–I**).

Preliminary searches of the NCBI nucleotide BLAST database showed a significant match between ITS sequences of the new species and core members of subtribe Tetraneurinae, including *Psilostrophecooperi* (A. Gray) Greene (88.69%), *Baileyamultiradiata* Harv. & A. Gray (88.89%), *Tetraneurisacaulis* Greene (88.48%) and *Hymenoxyslemmonii* Cockerell (88.68%). The ML phylogenetic tree, based on the ITS alignment from Baldwin et al. (2002), resolved the new species with very high (98 bs) support as nested in tribe Helenieae, where it was more closely related to *Psilostrophe* than other members of subtribe Tetraneurinae (Fig. 10). The new species + *Psilostrophe*, in turn, form the sister lineage to the clade containing *Amblyolepissetigera* DC., *Tetraneurisacaulis*, *Tetraneurisscaposa* (DC.) Greene, Hymenoxysambigensvar.floribunda (A.Gray) W.L. Wagner, *Hymenoxyshoopesii* (A. Gray) Bierne, and *Hymenoxyslemmonii* Cockerell. Addition of DNA sequence data (ITS) for five previously unsampled taxa of *Psilostrophe* resolves all currently recognised minimum-rank taxa in this genus as a monophyletic group separate from and sister to the new species. Amongst taxa of *Psilostrophe*, the narrowly endemic *P.bakeri* is resolved as sister to the rest of the genus, with *P.sparsiflora* next to diverge, followed by *P.cooperi*. Relationships amongst the highly-nested taxa *P.mexicana*, *P.gnaphalodes*, *P.tagetina* and *P.villosa* were not well supported with ITS data alone.

### ﻿Micro-anatomy

**Figure 11.**
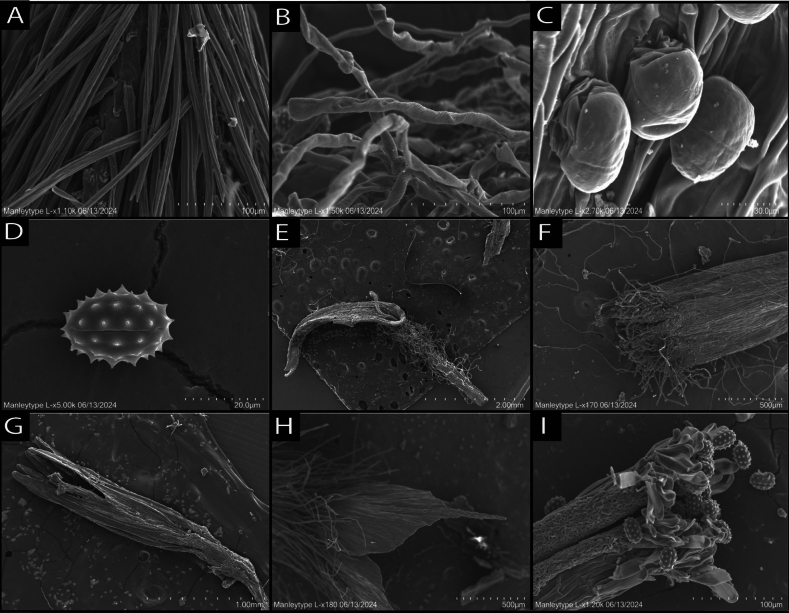
Scanning electron micrographs (SEM) of *Oviculabiradiata*. **A** Cypsela trichomes appear stiff, linear and end in a bifurcate (forked) tip **B** trichomes on leaf surface with a flexible, helical structure **C** short-stalked capitate glands on abaxial surface of ray corolla **D** pollen **E** ray floret without cypsela **F** disc corolla apex **G** anther column and exserted stigma **H** pappus palea tip with fine pleated serrations **I** style branch apex, with papillate trichomes sweeping pollen grains.

**Figure 12.**
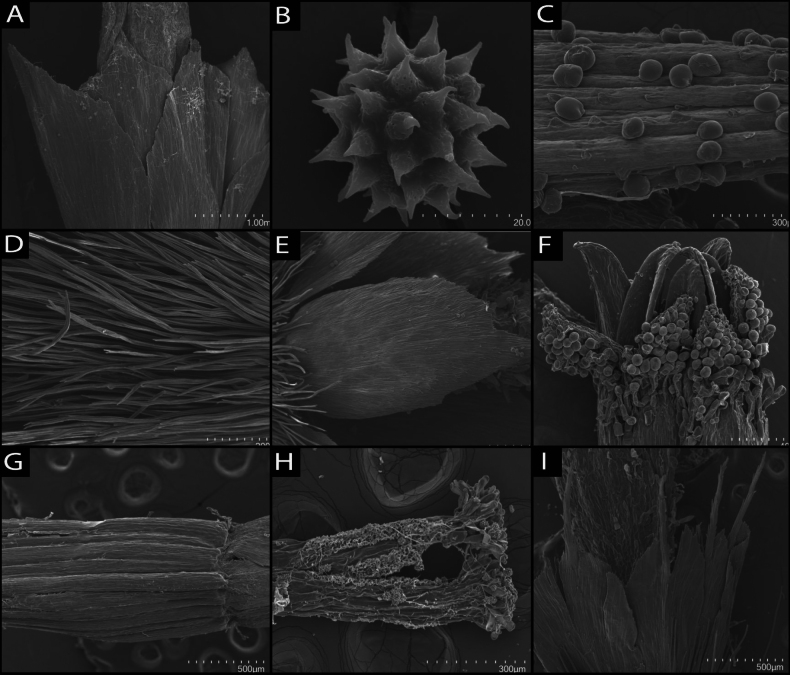
Scanning electron micrographs (SEM) of various genera of Tetraneurinae. **A** Pappus of *Amblyolepissetigera***B** pollen of *A.setigera***C** short-stalked capitate glands on disc corolla of *Baileyapauciradiata***D** stiff, twin hairs on cypsela of *Hymenoxyscooperi***E** hyaline, aristate palea-like pappus of *Hymenoxyscooperi***F** vesicular trichomes on abaxial surface of disc corolla lobes in *Psilostrophebakeri***G** ridges on the surface of a cypsela in *P.bakeri***H** sweeping papillate trichomes on style branch apices in *Tetraneurisscaposa***I** paleaceous pappus of *T.scaposa* with terminal, antrorsely setose bristle.

Micro-morphological features targeted using SEM for their value in evaluating phylogenetic relationships in the Heliantheae alliance included the surface texture of cypselae, pappus elements, trichomes, style trichomes, stigmatic surface, pollen shape and glands of vegetative and reproductive structures. A comparative table (Table 2) of these characteristics for genera of Tetraneurinae is given along with plates of SEM images (Figs 11, 12). Micro-anatomical features of the new species revealed by SEM include the dentate margins and pleated structure of hyaline pappus scales, style branch apices with terminal papillae and short stipitate glands that are present on the abaxial surface of ray and disc corolla lobes. Two types of trichomes were observed. Cypselae trichomes appear stiff, linear and end in a bifurcate tip. These conform with the cypselar trichomes observed in many other Compositae, also called twin hairs (“Zwillingshaare”) by Hess (1938). Trichomes on leaf tissues have a dilatated base (foot) that is notably wider than the rest of the structure, which has an elongated, flagellate body, an apex that ends in a simple, unbranched tip and a flexible, convoluted, helical structure, presumably giving the plant its characteristic woolly appearance. These trichomes conform to the oblique septate flagellate trichome type identified by Ramayya (1962), which occur in many groups of Compositae and often render the plant surface tomentose. Pollen grains of the new species measure approximately 20 micrometres in diameter and are oblate spheroidal in shape, with evenly spaced, symmetrical echinate projections.

**Table 2.** Comparison of morphology amongst genera of subtribe Tetraneurinae.

Character/taxon	*Hymenoxys*	*Tetraneuris*	*Amblyolepis*	*Psilostrophe*	*Ovicula*	*Baileya*
Life span	Annual, biennial or perennial	Annual or perennial	Annual	Biennial, perennial or shrubby	Ephemeral annual	Annual, biennial or perennial
Stems	5–120+ long; erect, often branched; glabrous or pilose	5–50 cm long; erect, or plants, acaulescent; sparsely to densely pilose	Usually 12–50 cm long; erect to decumbent; sparsely to moderately pilose	8–50+ cm long; spreading to erect; often densely woolly	1–4 cm long; prostrate; densely woolly	Usually 15–75 cm long; mostly erect; woolly
Leaves/blades	Basal and cauline; simple or 1–2-pinnately lobed; glabrous or pilose	Basal or basal and cauline; linear to lanceolate; glabrous or pilose	Cauline; linear to spatulate; pilose	Basal and cauline; linear to spatulate; sparsely to densely woolly	Basal ovate, involute to nearly folded; densely woolly	Basal and cauline; linear to ovate, often pinnately lobed; often densely woolly
Heads/peduncles	Single or several; peduncles 0.4–20+ cm long	Single or several; peduncles 0.5–40+ cm long	Usually single; peduncles to 20 cm long	Single or in clusters; peduncles 0.5–60+ cm long	Single; sessile or peduncles 1 mm long	Single or several; peduncles 2–12 cm long
Involucres	2.5–30 mm wide	6–20 mm wide	12–20 mm wide	2–7 mm wide	To 4–6 mm wide	5–25 mm wide
Phyllaries	2–3-seriate; sparsely to moderately pilose	3-seriate; sparsely to densely pilose	2-seriate; sparsely to moderately pilose	1–2-seriate; densely woolly	3-seriate; densely woolly	2-seriate; moderately to densely woolly
Ray florets	(3–)8–13+; corollas yellow to orange, corollas yellow to orange, nerves colourless or greenish; ray floret corollas 0.7–12 mm wide	None or 7–27; corollas yellow, nerves colourless, greenish, sometimes reddish-brown to maroon; ray floret corollas 2.5–6 mm wide	Usually 8–13; corollas yellow, nerves colourless or greenish, sometimes darker than background of laminae; ray floret corollas 4.5–10 mm wide	1–8; corollas yellow, nerves greenish, sometimes darker than background; ray floret corollas 3–20 mm wide	2 (-3); corollas whitish, nerves maroon; ray floret corollas 0.6–1 mm wide	5–7 or 20–55; corollas yellow, nerves colourless to greenish; ray floret corollas 4–7 mm wide
Disc florets	Usually 25–50+; corollas yellow to brownish-yellow, 1.5–7.4 mm long; pubescent distally, trichomes to 0.2 mm long	20–250+; corollas yellow, purplish distally, 1.6–3 mm long; pubescent mainly distally, trichomes to 0.1–0.2 mm long	20–-50; corollas yellow, 5–7 mm long; essentially glabrous distally	5–25+; corollas yellow to orange, 3.5–5.5 mm long; pubescent distally, trichomes 0.1–-0.2 mm long	10–12; corollas pale yellow, ca. 2–-3 mm long; tomentose distally, trichomes 0.3–0.5 mm long	10–20, or 40–100+; corollas yellow, 2.5–4 mm long; densely pubescent distally, trichomes to ca. 0.2 mm long
Cypselae	Obconic or obpyramidal, 1.4–4.7 mm long; glabrous or pilose	Obconic or obpyramidal, 1.5–4 mm long; moderately to densely tomentose	Obconic, 3–4.5 mm long, prominently 10-ribbed; ribs densely tan-tomentose	Cylindrical to clavate or obpyramidal, striate-ribbed, 2.5–4 mm long; glabrous, gland-dotted, or villous	Obconic-obpyramidal, faintly ribbed, 1.5–2 mm long; densely tomentose	Narrowly obpyramidal, 3–4 mm long, weakly ribbed or striate; glandular
Pappus	None or of 2–11(–15) usually aristate, obovate to lanceolate, scales, 0.8–4.3 mm long	Usually 4–8 aristate lanceolate, obovate, to oblanceolate, scales, 1–4.5 mm long	5–6 ovate scales 2–3.5 mm long	4–8 oblong, elliptic or lanceolate, scales, 1.5–3.2 mm long	5 aristate, oval scales, to 2 mm long	Usually absent, rarely scales
Base chromosome number	*x* = 15	*x* = 15	*x* = 19	*x* = 16	*x* = ?	*x* = 16

SEM images of representatives of related genera revealed similarities in the paleaceous and finely pleated structure of the pappus (Figs 11H, 12A, E, I), pollen (Figs 11D, 12D), short-stalked stipitate glands (Figs 11C, 12C), stiff cypselae trichomes with forked tips (Figs 11A, 12D), presence of the flexible helical trichomes that give the new species its woolly appearance (Fig. 11B) and style branch apices with sweeping papillae (Figs 11I, 12H). Some consistent differences that were noted between the new species and its sister genus *Psilostrophe* included the vestiture of disc and ray floret corolla lobes, which consist of papillae in *Psilostrophe*, whereas the new species possesses helical trichomes along ray throats and on abaxial surfaces of disc lobes (Figs 11F, 12F). Finer variability was evident in the size and shape of the apex in paleaceous pappus elements at shallower taxonomic scales (Figs 11H, 12A, E, I).

### ﻿Taxonomic treatment

#### ﻿ Ovicula biradiata

Taxon classificationPlantaeAsteralesCompositae

Manley, gen. et. sp. nov.

A67831A0-CC0A-5090-B27A-F6936DD1B820

urn:lsid:ipni.org:names:77356807-1

Figs 1 , 2 , 3 , 4 , 5 , 6 , 7 , 8

##### Description.

***Annuals***, small, flowering plants usually 1–2(–3) cm tall, from less than 1 cm wide to 3–7 cm across, whole plants densely white-tomentose. ***Root*** single, thread-like, 0.5–1 mm wide at the plant base. ***Stems*** unbranched, erect or branches, if evident, lateral, prostrate, spreading 1–4 cm in one or more directions, internodes ca. 1 cm long. ***Leaves*** basal, mostly in tight clusters or at nodes on short stems, proximal leaves spreading, distal ascending, petioles 1–4 mm long, blades ovate, 4–7 × 2.5–5 mm, entire, planar, gently involute or nearly conduplicate. ***Heads*** heterogamous, borne singly, essentially sessile (peduncles to ca. 1 mm long), obscured by woolly leaves. ***Involucres*** 5–7 × 4–6 mm, broadly funnelform to campanulate or subglobose. ***Phyllaries*** in 3 series, ca. 1–2 in outer series, ca. 1–2 in second series, outer slightly spreading, those in outer 2 series 3–4 × 2–3 mm, ovate, inner series ca. 7, linear, ca. 2 mm wide, with scarious margins ca. 0.5 mm wide, densely white-tomentose. ***Receptacles*** ca. 1 mm across, slightly saucer-shaped to flat, sometimes with a very small conic enation from near centre, otherwise basically smooth or with faint floret scars, epaleate. ***Ray florets*** 2(–3) per head, 3–6 × 0.6–1 mm long, positioned on opposing sides, pistillate and fertile, strap-like; corolla tube 2–3 mm long, densely pilose distally, with wavy trichomes 0.3–1 mm long, laminae 3–6 × 0.6–1 mm, 3-lobed, whitish, markedly 4-nerved proximally, 6-nerved distally, nerves maroon, proximal portion of the abaxial ray laminae densely covered with sessile or short stipitate glandular trichomes. ***Disc florets*** 10–12 per head, perfect and fertile; corolla pale yellow, ca. 2–3 mm long, tube 0.6–0.9 mm long, throat 1.6–1.8 mm long, lobes 5, 0.1–0.3 mm long, distalmost throat and lobes densely pilose with wavy trichomes 0.3–0.5 mm long; anthers yellow, distal anther appendage narrowly obovate to subsagittate; style tip appendage truncate, apex papillate. ***Cypselae*** of ray and disc florets similar, 1.5–2 mm long, obconic-obpyramidal, slightly compressed or obscurely 4–5-angled (prismatic), ribs 4–5, densely pubescent with straight, ascending-appressed, silvery trichomes 0.5–0.9 mm long, minutely forked at tip, partially obscuring the bases of pappus scales. ***Pappus*** of ray and disc florets similar, scales 5, ca. 1–3 × 0.8–1 mm, ovate, hyaline, with an apical arista ca. 1 mm long; the scales spreading when dry (Figs 1–7). Chromosome number unknown.

Similar to members of tribe Helenieae (sensu Baldwin et al. (2002)), especially *Tetraneuris*, with its annual habit, radiate heads, phyllaries in 3 series, convex, epaleate receptacles, ray florets pistillate and fertile, strap-like 3-lobed ray floret laminae with prominent, coloured veins, cypselae obconical, faintly ribbed and pappus of hyaline aristate scales, disc florets perfect and fertile, corollas yellow, 5-lobed; differs from other Helenieae genera by its smaller size, shorter stems, tightly clustered small leaves, greater tomentum density and smaller, sessile heads with only 2(–3) ray florets.

##### Type.

USA • Texas: Brewster Co.; Big Bend National Park, low gravelly limestone exposure, eroded alluvial flats, NE of Dagger Mt.; elev. 800 m, 20 Apr 2024, *Debra Manley 2*, with *C. Whiting*, *C. Hoyt*, *P. Manning*, and *S. Menzies*; holotype: SRSC 00058752 (BIBE 61799); isotype: CAS 1352777 (BIBE 61820).

***Paratypes.*** USA • Texas; Brewster Co.: Big Bend National Park, low gravelly limestone exposure, eroded alluvial flats, NE of Dagger Mt.; elev. 792.5 m, 20 Apr 2024, *Debra Manley 3*, with *C. Whiting, C. Hoyt, P. Manning*, and *S. Menzies*; BIBE 61800 (SRSC 00058751).

##### Etymology.

The generic name from Latin *Ovis* “sheep” and -*cula* (diminutive ending) references the dense woolly indumentum of this new plant. The name honours the desert bighorn sheep (*Ovis canadensis nelsonii*), an iconic, but threatened desert animal that is currently rebounding in this part of the Chihuahuan Desert, providing hope for other rare species like *O. biradiata*. The specific epithet *biradiata* references the typically two conspicuous ray florets, occasionally three per head, positioned on opposing margins of the capitulum (Figs 1, 4). A recommended common name for *O. biradiata* is “woolly devil”, in reference to the woolly indumentum, the proximity of populations to the locality known as Devil’s Den and the tendency for the ray florets to resemble horns.

##### Distribution and phenology.

*Oviculabiradiata* is known from limestone pediments of eastern Big Bend National Park where only three small populations have been found. Within these subpopulations, individual plants were abundant, but short-lived, indicating an ephemeral life history. The species was discovered on 2 March 2024 when plants were in full flower (Fig. 1). It is not presently known how early the plants may produce flowers, but, in the same general area, there are other species in several families that may bloom in early February or even earlier. By late May, after a period of warm and dry weather, the delicate annual plants had ceased vegetative growth and only desiccated inflorescences could be found (Fig. 4).

##### Habitat and associated taxa.

The general area of the three known locations for the new taxon, as so far observed, consists of a broad floodplain composed of fine sand and clay sediments and braided with drainage. This alluvial basin terrain is fringed with low, gravel-capped pediments which then extend into foothills and steeper slopes of a flanking limestone mountain range. The locations are within 625 m of each other and occur where a shallow layer of mixed alluvial gravel and stones overlie bedrock of the Boquillas Formation. This composite substrate occurs on both the Ernst and San Vicente members of the formation and the observed habitat exposures consist of thinly-bedded limestone, carbonate shale and siltstone overlain by Quaternary gravel, which is a heterogeneous mix of surrounding geologic substrates. One site includes a significant presence of iron-bearing rocks. The known locations receive full sun throughout the day with very little shade provided by the sparse vegetation or the flat topography (Figs 3, 4).

Widely-distributed species noted in the habitat include *Vachelliavernicosa* (Britton & Rose) Seigler & Ebinger, *Larreatridentata* (DC.) Coville, *Tiquiliagreggii* (Torr. & A. Gray) A.T. Richardson, *T.hispidissima* (Torr. & A. Gray) A.T. Richardson, *Agavelechuguilla* Torr., *Thymophyllaacerosa* (DC.) Strother, *Plantago* sp. L., *Oenothera* sp. L., *Physaria* sp. (Nutt.) A. Gray, *Nerisyreniacamporum* Greene, *Krameria* sp. Loefl., *Bouteloua* sp. Lag., *Aristida* sp. L., *Dasyochloapulchella* (Kunth) Willd. ex Rydb., *Ariocarpusfissuratus* K. Schum., *Echinocactushorizonthalonius* Lem., *Opuntia* sp. (L.) Mill. and *Grusoniaaggeria* (Ralston & Hilsenb.) E.F. Anderson. Cryptobiotic soil is present in the habitat as well. See Figs 2, 3 for habitat photos and Fig. 9 for a distribution map.

##### Conservation.

*Oviculabiradiata* is, so far, known only from within a small area in a seldom accessed part of Big Bend National Park. Nevertheless, the extremely narrow range and ephemerality of the species suggests that it is highly sensitive to variable weather patterns. Recently, this part of the Chihuahuan Desert has been under severe drought conditions and aridity is predicted to increase in this region due to climate change (Climate Change Response Program 2024). Under current IUCN guidelines for assessment of conservation status (IUCN Standards and Petition Committee 2022), *O.biradiata* would, therefore, preliminarily qualify as being vulnerable (VU) and under a high threat of extinction. More study is needed on the reproductive biology and population structure of *O.biradiata*, as well as potential threats to its habitat, to determine if the species should be listed by the U.S. Fish and Wildlife Service under the Federal Endangered Species Act. Due to the extreme sensitivity of the known collection sites the geocoordinates of the locality have been withheld and the locality is obscured on the map (Fig. 9).

## ﻿Discussion

The discovery of *Oviculabiradiata* underscores that the task of documenting and describing plant diversity is far from finished in the Chihuahuan Desert. Furthermore, that discoveries are not limited to unexplored or unpopulated regions and that interest and purposeful attention may still reveal novelties in places such as National Parks that might be considered “well-trodden” or fully understood. Encounters with novel plant species sufficiently different from their relatives to warrant description at generic rank are very uncommon in North America, but when they do occur, these often tend to be rare species associated with arid or edaphic micro-habitats where selection for unique growth forms is most pronounced (Stebbins 1952). Past examples of unique monospecific genera discovered in deserts or unique soils include *Apacheriachiricahuensis* C.T. Mason, *Dedeckeraeurekensis* Reveal & J.T. Howell, *Megacoraxgracielanus* S. Gonzalez & W. Wagner and *Yermoxanthocephalus* Dorn. Conservation management of *O.biradiata* will depend on gathering more detailed observations of its habitat specialisation, population size, reproductive biology, geographic range and life cycle and these are data that should be gathered with urgency. As drought conditions continue to increase in frequency and severity, opportunities to observe annual plants, including *O.biradiata*, may occur less frequently.

### ﻿Evolutionary implications

With the addition of *Oviculabiradiata*, subtribe Tetraneurinae contains six genera and 46 minimum rank taxa, making it the most diverse subtribe of Helenieae (Baldwin 2009). Extant diversity in this group is concentrated in western North America where they are distributed across a broad range of habitats from high mountains to low deserts (Baldwin and Wessa 2000). An ephemeral, annual life history has evidently evolved multiple times in this group apart from *O.biradiata*, as in *Baileyapauciradiata* Harvey & A. Gray, *Tetraneurislinearifolia* Greene and *Amblyolepissetigera* DC., *O.biradiata* stands out amongst other members of Tetraneurinae, however, for its minute stature, sessile heads and densely woolly foliage that effectively camouflages the plant into a background of coarse calcareous gravel. A salient, visually conspicuous characteristic of *O.biradiata* is its ephemeral ray florets, which usually appear in pairs (Figs 1–6).

### ﻿Phylogenetic relationships

Morphological features of *Oviculabiradiata* initially appeared to suggest a close link between the new genus and *Tetraneuris*, including maroon linear markings on the ray floret corollas (typically only visible on the abaxial face of the ray lamina in *Tetraneuris*), cypselae with a dense indument of fork-tipped trichomes and pappus of 4–6 hyaline, aristate scales. Some combination of these traits is present in other genera of subtribe Tetraneurinae, however, suggesting they may be shared ancestral characteristics. Molecular phylogenetic (ITS) data support a more distant relationship between *O.biradiata* and *Tetraneuris* than was expected from morphology and resolves the new genus as the sister lineage to the paper flowers (*Psilostrophe*). *Oviculabiradiata* bears resemblance to *Psilostrophe* in terms of its dense tomentose trichomes, leaves that are both basal and cauline and typically non-scapiform heads. ITS is a relatively easy-to-sequence DNA region that has been used for decades to resolve relationships at a variety of scales in Compositae, yet it represents only one line of genetic evidence. The possibility that conflicting relationships amongst genera of Tetraneurinae may be supported by alternative DNA regions or potentially reveal a role for other processes such as hybridisation in producing enigmatic evolutionary lineages like *O.biradiata*, are hypotheses that are worth exploring in future studies.

### ﻿Micro-anatomy

Micro-anatomical observations enabled by SEM revealed several characteristic features noted as diagnostic for the tribe (e.g. as Gaillardiinae in Robinson (1981)). These include a style apex with sweeping papillae, stigma with two receptive lines and oblate spheroidal pollen with regular echinate spines. Some characters revealed by SEM images for *O.biradiata*, include the pleated, serrate structural anatomy of the aristate pappus scales and foliar trichomes with a flagellate, helical body, which make up the plants woolly-tomentose indumentum. Short-stipitate glands present on the ray and disc corollas of *O.biradiata* resemble those found in many Compositae, which are often associated with sesquiterpene lactone synthesis (Robinson 2009). Phytochemical studies of Compositae, including members of tribe Helenieae, have yielded unique chemical compounds (e.g. Helenolins) with potential for anti-inflammatory and anti-cancer activity. The presence of short-stipitate glands in *O.biradiata* suggests this new species might contain secondary metabolites worthy of study for their potential medicinal value.

Finally, to encourage further study of this fascinating group, we present an updated key to the genera of Tetraneurinae, including *Ovicula*, based on information compiled from floras and observations of herbarium specimens in SRSC and CAS:

### ﻿Key to the genera of Tetraneurinae

**Table:** 

1	Phyllaries in 2 series, outer herbaceous, inner very short, hyaline, scale-like; cypselae 10-ribbed, the ribs densely tan-pubescent; herbage notably pleasant-scented; annuals; proximal leaves usually oblanceolate to broadly spatulate, semi-clasping, blades with long brownish trichomes, mostly on the margins	***Amblyolepis***
–	Phyllaries in 1–3 series, all herbaceous, inner not short, sometimes with scarious margins; cypselae 2–5-ribbed or angled, often weakly so, faintly striate in some taxa, the ribs or angles gland-dotted, naked or pubescent with long or short trichomes, these whitish or silvery; herbage lacking notable scent; annuals, biennials or perennials; proximal leaves of various shapes, not clasping, trichomes if present not long and brownish, instead white or colourless, often densely tomentose	**2**
2	Foliage glabrous or tomentellous (sparsely hairy), at the base often woolly, densely silky in *H.subintegra*; outer phyllaries usually partially connate; ray corollas ultimately withering and falling	***Hymenoxys***
–	Foliage densely woolly to tomentellous, sometimes glabrous; outer phyllaries distinct; ray corollas usually persistent in fruit, except readily dislodged in *Ovicula*	**3**
3	Ray florets 2(–3), corollas white with maroon nerves, readily dislodged from developing cypselae, laminae 0.6–1 mm wide; plants minute annuals, usually 1–2(–3) cm tall, 1–7 cm wide, branches if present lateral, prostrate, whole plants densely woolly; leaves crowded basally, entire, 4–7 mm long, 2.5–5 mm wide	***Ovicula***
–	Ray florets 1–55, rarely 0, corollas yellow or orange, with yellow or maroon nerves, usually persistent in fruit, laminae 0.7–20 mm wide; plants annuals, biennials or perennials, 5–100 cm tall (except 2–40 cm in several species of *Tetraneuris*), stems ascending to erect, scapiform in most *Tetraneuris*, glabrous or tomentellous to densely wololy; leaves basal or cauline, densely woolly or not, entire to pinnately lobed, 20–120 mm long, 5–30 mm wide	**4**
4	Pappus absent; leaf blades woolly, mostly 3-lobed or pinnate	***Baileya***
–	Pappus of 4–8 hyaline scales; leaf blades tomentellous to woolly, margins mostly entire, sometimes toothed or lobed	**5**
5	Ray florets 7–27, except none in *T.verdiensis*; disc florets 20–200+; plants tomentellous to somewhat woolly, scapiform, except stems erect in the annual *T.linearifolia*; heads mostly single	***Tetraneuris***
–	Ray florets usually 1–6; disc florets usually 5–17; plants woolly, not scapiform; heads single (in *P.cooperi*) or in clusters with peduncles 0.5–2.5 cm or more long	***Psilostrophe***

## Supplementary Material

XML Treatment for Ovicula biradiata

## ﻿Additional information

### Author ORCIDs

Debra L. Manley https://orcid.org/0009-0003-9345-4166

Isaac H. Lichter Marck https://orcid.org/0000-0003-3575-6003

Arturo Castro Castro https://orcid.org/0000-0002-2864-5180

Carolyn V. Whiting https://orcid.org/0000-0002-7665-1577

### Data availability

All of the data that support the findings of this study are available in the main text.
